# Ferritin Light Chain: A Candidate Autoantigen in Immuno-Related Pancytopenia

**DOI:** 10.3389/fimmu.2022.851096

**Published:** 2022-04-28

**Authors:** Yang Zhang, Shanfeng Hao, Na Xiao, Yu Zhang, Huaquan Wang, Lijuan Li, Rong Fu, Zonghong Shao

**Affiliations:** Department of Hematology, Tianjin Medical University General Hospital, Tianjin, China

**Keywords:** pancytopenia, antigenic epitopes, FTL, phage random peptide library, ELISPOT

## Abstract

The characteristic feature of immune-related pancytopenia (IRP) is autoantibody-mediated bone marrow (BM) damage and peripheral blood cytopenia. We found that the potential antigen of IRP was Ferritin light chain (FTL) by SEREX (serological analysis of recombinant cDNA expression libraries) in the previous study. In this study, we tried to explore the antigenic epitopes of FTL and verify its antigenicity in IRP. We found the possible FTL epitope: VNLYLQASYTYLSLG by phage random peptide library. Through ELISPOT, it was found that peptide VNLYLQASYTYLSLG can significantly stimulate the production of interleukin-4 and cannot stimulate the production of interferon-γ, which suggested that the peptide can obviously activate Th2 cells. Peptide–major histocompatibility complex tetramer elicited antigen-specific T cell responses. The expression levels of FTL were significantly increased in the patients with untreated IRP (P < 0.05). In conclusion, we found that FTL is the target antigen for some patients with IRP. The peptide of VNLYLQASYTYLSLG is an epitope of the target antigen. The target antigen is abnormally overexpressed on the membrane of BM cells, especially on the surface of CD34+ BM cells of patients with IRP. In addition, it is related to the severity of disease. These results provide a possible new target for the treatment of IRP in the future.

## 1 Introduction

Clinically, we often encounter a class of patients with multi-lineage cytopenia, and the diagnosis of these patients is often differentiated from a variety of hematologic diseases. Our previous research found that some patients with multi-lineage cytopenia did not fulfill the current diagnostic criteria of aplastic anemia (AA), myelodysplastic syndrome (MDS), paroxysmal nocturnal hemoglobinuria (PNH), etc. In the past, these patients were mostly treated according to atypical AA, MDS, refractory anemia, etc., but the efficacy was poor and long-term maintenance of transfusion was required. Therefore, it is of great significance to explore the characteristics of this part of unexplained cytopenia. Our research group focuses on the study of this part of the disease and has conducted systematic research on its pathogenesis, clinical manifestations, diagnosis and treatment, etc. The characteristic feature of the disease is autoantibody-mediated bone marrow (BM) damage and peripheral blood cytopenia. Corticosteroids combined with intravenous immunoglobulin therapy have a good therapeutic effect on these patients, and we named it immune-related pancytopenia (IRP) (1, 2). Later, Mufti also proposed an unexplained cytopenia (idiopathic cytopenia of undetermined significance, ICUS) (3, 4). Hence, we can also understand IRP as a special class of ICUS, namely, immune-related ICUS.

Over the past 20 years, our center has gradually explored the pathogenesis of IRP. We first studied the mechanism of blood cell destruction mediated by autoantibody Immunoglobulin G (IgG) and Immunoglobulin M (IgM) (5) and then found the presence of CD4^+^ T cell hyperfunction in patients with IRP (6). Further studies found that abnormalities of pDC, Th17, Tfh, Treg, Th9, and Breg cells also play different role in the pathogenesis of IRP (7–12). However, the target antigens on the surface of hematopoietic cells targeted by autoantibodies in patients with IRP remain unclear. Searching for target antigens is of great significance for better understanding the pathogenesis, differential diagnosis, and diagnosis and treatment of IRP. To clarify the target autoantibodies and antibody-special antigen, we constructed a recombinant cDNA expression library and combined it with serological analysis, and we screened seven proteins including Ferritin light chain (FTL) that may be the target antigens of IRP (13). Then, by indirect Enzyme-Linked Immunosorbent Assay (ELISA) and Quantitative Real-Time PCR (Q-PCR), we found that serum FTL antibody and FTL-mRNA expression levels were upregulated in patients with IRP (14), indicating that FTL plays an important role in the pathogenesis of IRP.

Ferritin, the main form of stores iron in the body, serves a crucial role in regulating iron homeostasis and protecting cells against oxidative damage during inflammation and oxidative stress. It consists of two subunits, the ferritin heavy chain (FTH; 21 kDa) and FTL (19 kDa). Iron is essential for innate host defense mechanism and acquired immune responses, and a number of studies have shown that abnormalities in ferritin are closely related to immune abnormalities associated with autoimmune diseases. Systemic lupus erythematosus (SLE) is a type of autoimmune disease. The level of ferritin shows a positive correlation with the inflammation state and the severity of anemia in patients with SLE (15). Another study proposed that overexpression of FTL leads to significant reduction of LPS-induced inflammatory factor. On the contrary, FTL knockout showed the opposing results (16). It is supposed that FTL may play an immunomodulatory role in response to LPS in murine macrophages.

Therefore, on the basis of preliminary study that FTL may be the target antigen of IRP by using SEREX (serological analysis of recombinant cDNA expression libraries) method, this study intends to explore the antigenic epitope of FTL and verify its antigenicity in IRP. It will provide a possible new target for the diagnosis and treatment of IRP.

## 2 Materials and Methods

### 2.1 Patients

A total of 23 patients with untreated IRP (median age, 44; range, 20–72 years old) and 10 healthy blood donors as normal controls (median age, 34; range 25–72 years old) were selected, and their serum was collected for purification of antibodies. Meanwhile, PBMCs (peripheral blood mononuclear cells) were extracted aseptically from these patients’ blood and stored in −80°C refrigerator for future ELISPOT experiment. The serum samples of the ELISA experiment from 20 patients with untreated IRP with a median age of 48 (range, 13–69) years old, 14 recovered patients with IRP with a median age of 40 (range, 19–72) years old, and 14 normal controls with a median age of 35 (range, 23–69) years old.

In addition, 50 samples, from which 17 patients with untreated IRP (median age, 63; range, 15–78 years old), 20 recovered patients with IRP (median age, 48; range, 16–78 years old), and 13 case-control group (median age, 52; range, 32–83 years old), were used for Q-PCR.

In the flow cytometry (FCM) experiment, there were 15 patients with untreated IRP with a median age of 53 (range from 22 to 73) years old and 12 recovered patients with IRP with a median age of 47(range from 7 to 82) years old. Eleven patients (median age, 64; range, 17–72 years old) in the case-control group were analyzed by FCM. We further detected the level of FTL on the surface of CD34^+^, CD235a^+^, and CD15^+^ BM cells. Eight patients with untreated IRP (median age, 35; range, 12–53 years old), 10 remission patients with IRP (median age, 47; range, 7–76 years old), five patients of AA and low-risk MDS (median age, 55; range 19–80 years old), and five case controls (median age, 38; range 23–76 years old) were enrolled in this study.

Written informed consent was obtained from all participants and the protocol was approved by the Ethics Committee of the General Hospital of Tianjin Medical University. All the experiments were carried out according to the approved protocol. All patients met diagnostic criteria for IRP (2), exclusion of other potential causes of pancytopenia such as AA, MDS, and PNH.

### 2.2 Selection of Peptides From a Phage-Displayed Random Peptide Library and Synthesizing Antigen Peptides

Serum samples were divided into two groups: normal controls and patients with untreated IRP. A total of 0.5 ml was taken from each sample and then mixed them separately in each group. Antibodies were purified by affinity purification. The concentration of antibodies was determined by A280, and purity was assessed by Sodium Dodecyl Sulfate Polyacrylamide Gel Electrophoresis (SDS-PAGE). Then, we used phage-displayed random peptide library (Ph.D-7 Phage Display Peptide Library Kit, New England Biolabs) for biopanning according to the kit’s recommendations. The procedure for the experiment was as follows: 10 μg of patient’s serum were coupled to 10 µl of ProA/G magnetic beads overnight at 4°C and 200 µg of normal control’s serum were conjugated to 50 µl of ProA/G magnetic beads overnight at 4°C. The supernatant was carefully discarded with the gun and gently rinsed with PBS for three times. Both groups were added into 1 ml of 4% PBSM and incubated at room temperature for 1 h. The supernatant was carefully discarded and gently washed with PBS for one time. Peptide library (10 μl) with a titer of 1.0 × 10^11^ pfu/ml was diluted into 1 ml of 4% PBSM and added into control group magnetic beads, which were reacted at room temperature for 1 h. The obtained solution was placed into the magnetic beads of the experimental group and placed at room temperature for 2 h. PBST (0.1%) and PBS were added and washed five times each, and the washing times of each round gradually increased. The bound phages were eluted by 100 μl of 0.2M Gly-HCl (pH 2.2) for 8 min and neutralized them with 15 μl of 1 M Tris-HCl (pH 9.1). Neutralization solution (10 μl) was diluted for 1,000, 10,000, or 100,000 times, and then, 200 μl of *E. coli* (ER2738) was infected for 5 min at 37°C. Three milliliters of 50°C top layer medium was added to the solution. The mixture was titered on LB/IPTG/Xgal plates overnight at 37°C. We counted blue plaques on the plates and the number of recovered phages. The eluted phages infected *E. coli* (ER2738) for 4.5h at 37°C. After centrifugation at 12000g for 10 min, we put the supernatant into a 1/5 volume of 20% PEG/2.5M NaCl for 2h at 4°C. After centrifugation again, the solution was removed and the pellet was resuspended in the medium. We repeated above steps for the second and third rounds of biopanning, and the number of washing steps was raised by five each round. After the third round of screening, the final eluted phages infected *E. coli.* Three milliliters of 50°C top layer medium was added to the solution. The mixture was titered on LB/IPTG/Xgal plates overnight at 37°C. The single blue plaques were picked up randomly and seeded into a 96-well plate for 4.5 h at 37°C. Then, the solution was centrifuged to get the phage. Antibodies of normal controls and patients, which were diluted with CB to a final concentration of 1 µg/ml, were added to a 96-well plate (100 μl per well) overnight at 4°C. Blocked with 4% PBSM at 37°C for 1 h. The blocking solution was removed and then washed in PBS once. Fifty microliters of 4% PBSM and 50 μl of obtained phage were added into each well for 1 h at 37°C, and then, the plate was washed three times with PBS and three times with PBST. Anti-p8/HRP antibody was added and incubated at 37°C for 1 h, and then, the plate was washed three times with PBS and three times with PBST. TMB substrate (100 μl per well) was added, and the reaction was terminated after 15 min avoiding light. Readings were taken using a microplate reader. Positive clone plaque was amplified for subsequent sequencing. The sequencing results of the positive clones were compared with the amino acid sequence of the FTL. We extended the antigen peptides by T cell epitope prediction software TepiTool and synthesized the antigen peptide VNLYLQASYTYLSLG by GenScript company, China.

### 2.3 Isolation and Frozen of PBMCs

Lymphocyte separation tubes (DAKEWE, China) were used to isolate PBMCs from whole blood of newly diagnosed patients with IRP. According to the instruction of the kit, we cryopreserved PBMCs in the refrigerator at −80°C using a freezing kit (DAKEWE, China).

### 2.4 IL-4 Elispot Assay

According to the supplier’s instructions, the Elispot was conducted using the human interleukin-4 (IL-4)–precoated Elispot kit (MABTECH, Sweden). Briefly, we resuscitated the cells and examined cell survival rate. The reagent dissolved the peptide according to the instructions of the peptide manual. Then, peptides were further diluted to the desired concentration using serum-free medium (DAKEWE, China). After preparation and blocking of plate, we added the stimuli (50 μM peptide) followed by the PBMC suspension (5 × 10^5^cells per well) in experimental wells (two wells per experimental group). The plate was incubated in a 37°C 5% CO_2_ incubator for 12–48 h. The cells were removed, and diluted IL-4-II-ALP (1:300, 100 μl per well) was added and incubated for 2 h at room temperature. After washing with PBS, we added a substrate solution of 100 μl per well. Until spots emerge, we washed the plate with tap water and then count spots.

### 2.5 IFN-γ Elispot Assay

Elispot was performed using the human interferon-γ (IFN-γ)–precoated ELISPOT kit (DAKEWE, China) as suggested by the guidelines for users. The experimental step is similar to the previous paragraph. Key steps are as follows. We put the mixture of stimuli (50 μM peptide) and PBMC suspension (5 × 10^5^cells per well) into experimental wells. The plate was incubated in a 37°C 5% CO2 incubator for 12–48 h. Biotin-labeled antibody against IFN-γ was added to each well and incubated at 37°C for 1 h. Enzyme-labeled avidin (100 μl per well) was added and incubated at 37°C for 1 h. Finally, 100 μl per well of AEC substrate solution was added to the plate. Until spots emerge, we washed the plate three times with tap water and then count spots.

### 2.6 MHC-Peptide Tetramers Analysis

The top three HLA-DR alleles found in the China population are DRB1*09:01 (14.62%), DRB1*15:01 (10.49%), and DRB1*07:01 (5.90%). In addition, in a number of studies, the HLA class II DRB1 antigen DR15, especially HLA-DRB1*1501, is associated with the pathogenesis of AA. HLA-DRB1*1501 is significantly higher in patients with SAA (severe AA) than normal controls (17–19). Thus, we selected to synthesize HLA-DRB1*15:01 tetramer. Major histocompatibility complex (MHC)–peptide tetramer complexes (PE-labeled ProT2 MHC-VNLYLQASYTYLSLG class II tetramer, HLA-DRB1*15:01) were purchased from ProImmune company, and the experiment was conducted according to the instructions. The ProT2 MHC class II tetramer was placed in a chilled microcentrifuge and centrifuged at 14,000g for 5 min. Multiplex sequence–specific primers (PCR-SSP) were used to detect HLA-DRB1 genotyping, and steps and primer sequences were detailed as previously reported (20). We divided the patients with untreated IRP into HLA-DRB1*15:01 group and non–HLA-DRB1*15:01 group for follow-up experiments. We extracted and counted the PBMCs of the patients and normal controls (HLA-DRB1*15:01) and put about 1× 10^6^ to 2 × 10^6^ cells in each tube. The cells were washed twice and resuspended in 50 μl of residual liquid. Five microliters of PE-labeled MHC class II tetramer was added, incubated at 37°C in the dark for 2 h, and then washed again. CD4-FITC (BD Biosciences, USA) and CD3-PerCP (BD Biosciences, USA) antibodies were added, incubated on ice for 30 min in the dark, washed twice, and stored in the fix solution (1% fetal calf serum, 2.5% formaldehyde in PBS) in the dark. FCM was used to detect the expression of peptide-specific T lymphocytes.

### 2.7 Flow Cytometric Analysis of FTL

We firstly analyzed the expression levels of FTL on the membrane of BM mononuclear cells (BMMNCs) by FCM. We take 100 μl of BM from patients with IRP and case controls separately. Five microliters of mouse anti-human FTL-PerCP (Novus Biologicals, USA) was added to the experimental tube, and 5 μl of the anti-mouse IgG1-PerCP (BD Biosciences, USA) isotype control was added to the control tube. After shaking with the oscillator, tubes were put in a 4°C refrigerator and react for 30 min in the dark. Hemolysin (3ml; BD PharMingen, USA) was added to each tube, shake well, and reacted for 10 min in the dark at room temperature. Then, we centrifuged the tubes at 1,500 r/min for 5 min, discarded the supernatant, washed them twice with PBS, added 300 μl of PBS, and detected the FTL expression level by FACS Calibur flow cytometer (BD Biosciences). We further analyzed the expression levels of FTL on the membrane of CD34^+^ BM cells, CD15^+^ BM cells, and CD235a^+^ BM cells. The steps were performed as we described above. The primary reagents were used: anti-human FTL-PerCP (Novus Biologicals, USA), anti-human CD34-FITC (Quantobio, China), anti-human CD15-FITC (BD Biosciences, USA), and anti-human CD235a-FITC (BD Biosciences, USA).

### 2.8 ELISA

We purchased a human FTL ELISA kit (Sino Biological Inc., China) to detect FTL levels in plasma samples. All steps were performed in accordance with the guidelines for users. The level of FTL in plasma is expressed in nanograms per milliliter. Each independent experiment was performed two times.

### 2.9 Isolation of Bone Marrow Mononuclear Cells

Fresh heparinized BM samples were diluted with phosphate-buffered saline (PBS) in a 1:1 proportion. The same volume of lymphocyte separation solution (Solarbio, China) as the mixture was added to the centrifuge tube. The diluted BM samples were carefully layered on lymphocyte separation solution and centrifuged at 400g for 20min. A clean pipette was used to transfer the lymphocyte layer to a clean centrifuge tube and then further washed with PBS. The supernatant was discarded. Red blood cell (RBC) lysis solution (Solarbio, China) was added into the tube and incubated for 8 min away from light, centrifuged, and washed with PBS again. We obtained BMMNCs for follow-up experiments.

### 2.10 Quantitative Real-Time PCR

RNA extraction from BMMNCs and reverse transcription was described previously (14). The sequence of primers was as follows: FTL: forward, 5′-CAGCCTGGTCAATTTGTACCT-3′ and reverse, 5′-GCCAATTCGCGGAAGAAGTG-3′, and GAPDH: forward, 5′-CCGGGAAACTGTGGCGTGATGG-3′ and reverse, 5′-AGGTGGAGGAGTGGGTGTCGCTGTT-3′. SuperReal PreMix Plus (SYBR Green) (Tiangen Biotech Co., Ltd.China) was used for quantitative real-time PCR. The amplification conditions were as follows: Cycle 1: (1×) Step 1: 95.0°C for 15 min; Cycle 2: (45×) Step 1: 95.0°C for 10 s, Step 2: 55.0°C for 32s. Cycle 3: (51×) Step 1: 70.0°C–95.0°C for 30 s. Cycle 4: (1×) Step 1: 4.0°C for 10 s. We used 2% agarose gel electrophoresis to verify the size of PCR product predicted from the cDNA. The Q-PCR results was analyzed by 2^−ΔΔCt^ method (21). The Ct value was defined as the number of PCR cycles required for the reporter fluorescence reached a threshold.

### 2.11 Western Blot

Western blot was performed as described previously (22). The primary reagents were used: rabbit anti-FTL (dilution, 1:1,000; Abcam, UK), rabbit anti-GAPDH antibodies (dilution, 1:1,000; Cell Signaling Technology, Inc., USA), horseradish peroxidase (HRP)–conjugated anti-rabbit IgG (dilution, 1:2,000; Cell Signaling Technology, Inc., USA), ECL Chemiluminescent HRP Substrate (Merck Millipore, USA), and BCA Protein Assay Kit (Beijing Biosynthesis Biotechnology Co., China). The gray levels of bands were analyzed by ImageJ software. The relative gray level was represented by relative IntDen (relative IntDen = the integrated band density (IntDen) of FTL/IntDen of GAPDH).

### 2.12 Statistical Analysis

All the data were expressed by mean ± standard deviation and analyzed by non-parametric tests. SPSS version 25.0 (SPSS, Inc., USA) was used. *P* < 0.05 was considered to indicate a statistically significant difference.

## 3 Results

### 3.1 To Screen FTL Epitopes and Synthesize Antigen Peptides

#### 3.1.1 The Purification of Antibody

**Figure 1:**
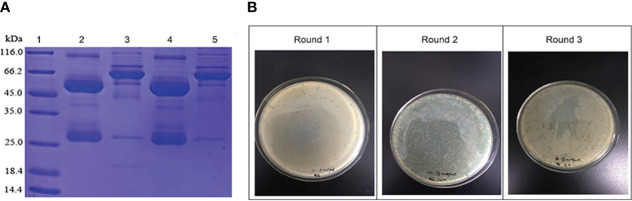
**(A)** The purified antibody of normal controls (lane 2) and patients with untreated IRP (lane 4) assessed by SDS-PAGE. Lane 1, molecular weight markers. Lanes 3 and 5, only a small amount of antibody remained in the flow-through solution of normal controls (lane 3) and patients with untreated IRP (lane 5). **(B)** Phage biopanning and enrichment after three rounds.

The antibody purity of patients with untreated IRP and normal controls was assessed by SDS-PAGE (**Figure 1A**). It can be seen from the gel picture that after purification, human antibodies were obtained in the eluate. Only a small amount of antibody remained in the flow-through solution (after mixing the sample with 1% sodium acetate buffer at a 1:4 volume ratio, the mixture passing through the column was defined as flow-through solution), and the purification experiment obtained the expected experimental results. Because of the relatively large sample load, you can see the appearance of the hybridized band. Considering that Protein G magnetic beads will be used in the subsequent screening process, which is equivalent to purifying the antibody again, no further processing of the sample.

#### 3.1.2 Biopanning

After three rounds of panning, the titers of phage gradually increased, and the phage was obvious enriched (**Figure 1B**). The experiment was in line with expectations, and the next step was to pick single phage spot for ELISA detection.

#### 3.1.3 Identification of Positive Clones

Through the first ELISA experiment, it was found that the value of most positive wells was significantly higher than that of negative control wells. Hence, subsequent ELISA experiments could be carried out. We selected single phage plaques of two plates for the second ELISA experiment. Considering that we need to screen phage clones that can recognize the patient’s antibody but cannot recognize normal controls’ antibody. The same phage single spot was reacted with the antibody of the patient and the normal control, respectively. By comparing the reaction results, the 10 clones were selected and sent for sequencing analysis. The sequencing results are shown in **Table 1**.

**Table 1:** The results of sequencing.

Clone	Nucleotide sequences	Peptide sequences
1D2	TGGAGTCTTGGGTATACTGGG	WSLGYTG
1A5	TGGAGTCTTGGGTATACTGGG	WSLGYTG
2C11	ACGATTTATACTACTTGGCAG	TIYTTWQ
2C9	TGGAGTCTTGGGTATACTCGG	WSLGYTR
2E9	ACGATTTATACTACTTGGCAG	TIYTTWQ
2A9	ACGATTTATACTACTTGGCAG	TIYTTWQ
2B8	TATACGACTACTTTGACGTAT	YTTTLTY
2B12	ACGATTTATACTACTTGGCAG	TIYTTWQ

After sequence analysis, a total of four different clones were obtained. Comprehensive analysis of these four sequences revealed that all peptides contained two amino acids YT. The sequence of the polypeptide was further compared with the FTL amino acid sequence, and the results are as follows:

1D2: WSLGYTG

2C11: TIYTTWQ

2C9: WSLGYTR

2B8: YTTTLTY

FTL antigen sequence:

MSSQIRQNYSTDVEAAVNSLVNLYLQASYTYLSLGFYFDRD802665802665DVALEGVSHFFRELAEEKREGYERLLKMQNQRGGRALFQDIKKPAEDEWGKTPDAMKAAMALEKKLNQALLDLHALGSARTDPHLCDFLETHFLDEEVKLIKKMGDHLTNLHRLGGPEAGLGEYLFERLTLKHD.

Through sequence alignment, it was found that two amino acids YT were also found in FTL antigen, and two clones 1D2 and 2C9 also had SLG sequences, which were consistent with the SLG sequences in the antigen sequence. Therefore, after screening peptides by antibodies from patients and normal controls, a peptide sequence that can recognize the antibodies in patients’ serum but not in normal controls’ serum was obtained. The antigenic epitope recognized by the antibody in the patient’s serum may be the sequence YTYLSLG.

#### 3.1.4 Antigenic Peptides Were Prolonged Using T Cell Epitope Prediction Software

The epitopes that can specifically bind to T lymphocytes are called T cell epitopes. T cell epitopes are linear epitopes, which are peptide chains composed of sequentially connected amino acids. T cell epitopes can be divided into CD4^+^ and CD8^+^ T cell epitopes. Previously, our research group found that patients with IRP had the imbalance ratio of Th1/Th2 cell and hyperfunction of Th2 cells. Therefore, it is of vital importance to study CD4^+^ T cell epitopes in patients with IRP. However, antigen cannot be directly recognized by antigen-specific T lymphocytes. It can only be recognized when it is combined with MHC molecules. After the antigen is processed by antigen presenting cells, the antigenic peptide is presented to T cell receptor (TCR) on the surface of CD4^+^ T cells by MHC class II molecules. Such antigenic short peptide with immune activity is called CD4^+^ T cell epitope. The FTL antigen peptide that we found is a heptapeptide, and the antigen peptide that MHC class II molecules can bind is 13–17 amino acids, so we need to extend the peptide.

How to lengthen peptides? As a linear epitope, T cell epitope can present a variety of peptide combinations. Hence, it takes a long time to find only a limited number of peptides by using traditional experimental methods. T cell epitope prediction software can increase the efficiency of discovering new epitopes by 10–20 times and can reduce the experimental workload by up to 95%. It has become an important tool for epitope positioning. In this study, TepiTool, a T cell epitope prediction software in the immune epitope database (IEDB), was used to predict the antigen epitope (15 peptide) of FTL that can bind to MHC class II molecules (23–27). Compared with the YTYLSLG sequence found by phage random peptide library, the peptide was extended. GenScript company (Nanjing, China) was commissioned to synthesize positive peptide 1: VNLYLQASYTYLSLG and negative peptide 2: IKKPAEDEWGKTPDA for subsequent experiments.

### 3.2 To Study the Effect of Antigen Peptide by Using Elispot and MHC-Peptide Tetramers Method

#### 3.2.1 IL4-Elispot

**Figure 2:**
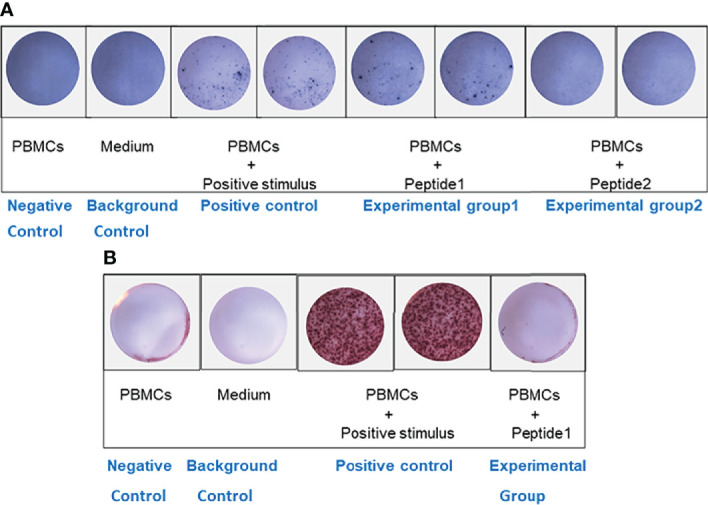
ELISPOT analysis. **(A)** IL-4 ELISPOT. The number of spots in experimental group 1 (PBMCs + peptide 1) was significantly higher than that in the negative control well. However, there was no difference in the number of spots in experimental group 2 (PBMCs + peptide 2) compared with the negative control well. **(B)** IFN-γ ELISPOT. There was no difference in the number of spots between the experimental well (PBMCs + positive peptide 1) and the negative control well.

It was found that the number of spots in experimental group 1(PBMCs + peptide 1) was significantly higher than that in the negative control well (only PBMCs were added), indicating that PBMCs stimulated by peptide 1: VNLYLQASYTYLSLG could significantly activate Th2 cells and secrete cytokine IL-4, which could be used as a positive peptide for subsequent experiments. However, there was no difference in the number of spots in experimental group 2 (PBMCs + peptide 2) compared with the negative control well (**Figure 2A**), indicating that peptide2: IKKPAEDEWGKTPDA could not activate Th2 cells to secrete cytokine IL-4, which was in line with the standard of negative peptide.

#### 3.2.2 IFN-γ Elispot

To further verify the effect of positive peptide 1: VNLYLQASYTYLSLG that we screened on Th1 cells, we also put PBMCs into IFN-γ Elispot plate. There was no difference in the number of spots between the experimental well (PBMCs + positive peptide 1) and the negative control well (**Figure 2B**), indicating that peptide1: VNLYLQASYTYLSLG could not activate Th1 cells to secrete cytokine IFN-γ.

### 3.3 Antigen-Peptide-Specific T Cells Are Detectable by Using MHC-Peptide Tetramers

**Figure 3:**
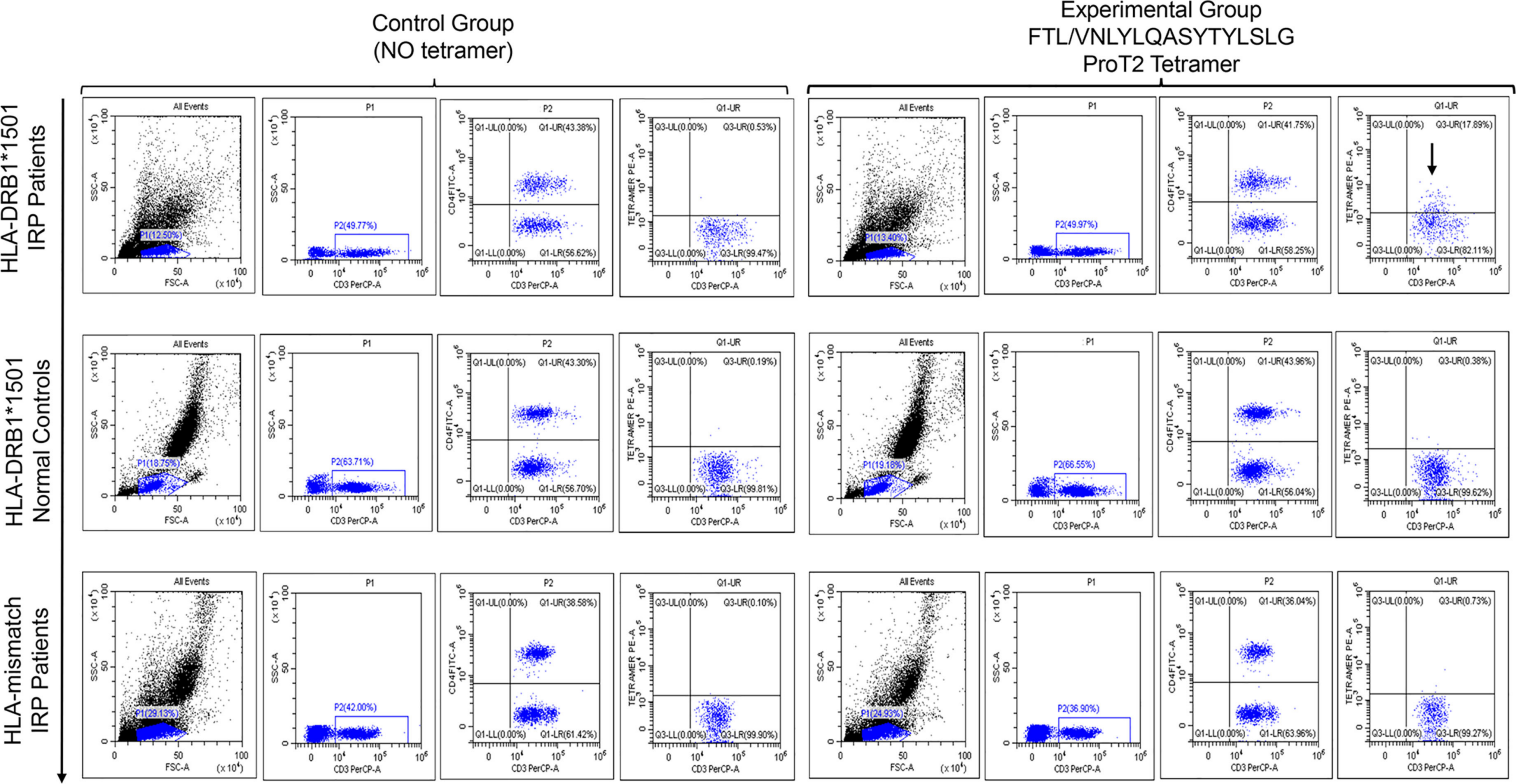
Peptide/HLA-DRB1*15:01 MHC class II tetramer staining for antigen-specific CD4^+^ T cells analyzed by flow cytometry. The top row plots show PBMCs from patients with untreated IRP (HLA-DRB1*15:01). The image in the middle row shows sample of cells from HLA-DRB1*15:01 normal controls. The bottom row figure shows cell samples from patients with untreated IRP (non–HLA-DRB1*15:01). All plots were derived by gating on CD3-postive and CD4-postive lymphocytes. The first four images in each row are the control group (no tetramer), and the last four ones are the experimental group with tetramer. In the upper right quadrant of the plot, there is a population of antigen-specific CD4^+^ T cells is visible from cells of patients with untreated IRP (HLA-DRB1*15:01) stained with PE-labeled ProT2 MHC tetramer compared to the control group (black arrows).

We stained PBMCs from patients with untreated IRP (HLA-DRB1*15:01 and non–HLA- DRB1*15:01) and normal controls (HLA-DRB1*15:01) with PE-labeled ProT2 MHC-VNLYLQASYTYLSLG class II tetramers (**Figure 3**). It can be observed from the pictures that there is a population of antigen-specific CD4^+^ T cells, which is visible from cells of patients with untreated IRP (HLA-DRB1*15:01) stained with PE-labeled ProT2 MHC tetramer compared to the control group. This result indicates that the epitope VNLYLQASYTYLSLG derived from FTL as potential antigens to boost the antigen-specific CD4^+^ T cell production.

### 3.4 To Study the Expression Levels of FTL in Patients with IRP, and the Correlation Between the Expression Level of FTL and Clinical Indicators

#### 3.4.1 The Expression Levels of FTL on the Membrane of BM Cells by Flow Cytometry

**Figure 4:**
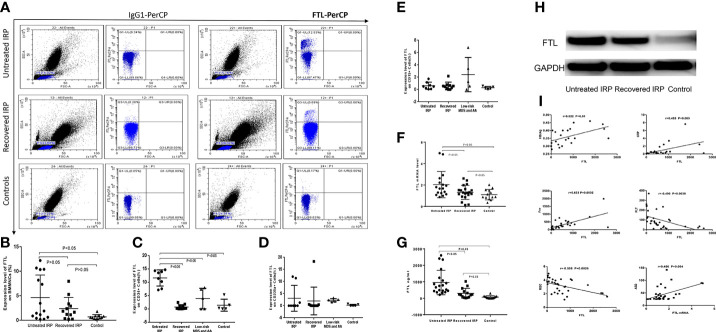
Analysis of FTL levels in patients with IRP, and the correlation between the expression level of FTL and clinical indicators. **(A)** Flow cytometry plots of FTL. **(B)** Results of the FCM assay of FTL on surface of BMMNCs. **(C)** Results of the FCM assay of FTL on surface of CD34^+^ BM cells. **(D)** Results of the FCM assay of FTL on surface of CD235a^+^ BM cells. **(E)** Results of the FCM assay of FTL on surface of CD15^+^ BM cells. **(F)** Q-PCR analysis of FTL-mRNA. **(G)** FTL expression level was analyzed by ELISA. **(H)** Western Blot analysis of FTL protein levels. **(I)** The correlation between the expression level of FTL and clinical indicators.

FCM analysis of FTL was shown in **Figure 4A**. The expression levels of FTL on the membrane of BMMNCs were (4.62 ± 4.61), (2.36 ± 2.30), and (0.68 ± 0.46) in the untreated group, the recovered group, and the case-control group, respectively. Compared with the case-control group, FTL level in the untreated IRP group was significantly increased (*P* < 0.05) (**Figure 4B**). There was no significant difference between the other two groups (*P* > 0.05).

To further clarify the specific BM cells with increased FTL expression, we further studied the expression levels of FTL on the membrane of CD34^+^, CD15^+^, and CD235a^+^ BM cells, respectively. The expression levels of FTL on the membrane of CD34^+^ BM cells in the untreated group (11.61 ± 3.03%) were significantly higher than that of the recovered group (0.71 ± 0.86%, *P* < 0.05), the group of AA and low-risk MDS (3.88 ± 3.84%, *P* < 0.05), and the case-control group (1.39 ± 2.26%, *P* < 0.05). There were no significant differences among the other three groups (*P* > 0.05) (**Figure 4C**). The expression levels of FTL on the membrane of CD235a^+^ BM cells were (3.11 ± 5.39%), (2.41 ± 6.42%), (2.29 ± 0.67%), and (0.18 ± 0.36%) in the untreated group, the recovered group, the group of low-risk MDS and AA, and the case-control group, respectively. There were no significant differences among these four groups (*P* > 0.05) (**Figure 4D**). In addition, there were no statistical differences among the expression levels of FTL on the membrane of CD15^+^ cells in the untreated group (0.62 ± 0.58%), the recovered group (0.57 ± 0.66%), the group of low-risk MDS and AA (2.43 ± 2.79%), and the case-control group (0.28 ± 0.22%), *P* > 0.05 (**Figure 4E**).

#### 3.4.2 The Expression Levels of FTL-mRNA by Q-PCR

The expression levels of FTL-mRNA in the BMMNCs of the untreated group, the recovered group, and the case-control group were (2.04 ± 1.22), (1.31 ± 0.66), and (1.10 ± 0.49), respectively. Compared with the case-control group, we observed a significant increase of FTL-mRNA in the untreated IRP group (*P* < 0.05) (**Figure 4F**). We did not observe significant difference between the recovered group and the case-control group (*P* > 0.05).

#### 3.4.3 The Levels of Serum FTL Antigen in Patients With IRP by ELISA

ELISA results showed that the levels of serum FTL in the untreated IRP group (954.85 ± 743.34) ng/ml and recovered IRP group (303.22 ± 300.79) ng/ml detected by ELISA were higher than the normal control group (70.46 ± 82.63) ng/ml; the difference was statistically significant (*P* < 0.05) (**Figure 4G**).

#### 3.4.4 FTL Expression Level Was Measured Using Western Blot

The Western blot results are shown in **Figure 4H**. The relative gray value of FTL/GAPDH in the untreated IRP group was 0.872 and in the recovered group was 0.659. The relative gray value of FTL/GAPDH is 0.301 in the control group. The expression level of FTL in the patients with untreated IRP was higher than that of recovered IRP group and case-control group.

#### 3.4.5 Correlation Analysis Between FTL Level and Clinical Indicators

The following data were collected for most of the subjects: blood routine (33 cases), ferritin (29 cases), Anti streptolysin O (ASO) (33 cases), Epstein-Barr virus (EB virus) (17 cases), hepatitis B (22 cases), erythropoietin (EPO) levels (16 cases), etc. FTL levels detected by ELISA were negatively correlated with RBC and Platelets (PLT) and positively correlated with ferritin, CRP, and HBeAg. The FTL-mRNA levels were positively correlated with ASO (**Figure 4I**).

## 4 Discussion

IRP is a type of autoimmune disease caused by the binding of anti-BM hematopoietic cell autoantibodies to the target antigen, resulting in a decrease in blood cells. It is of great importance to find its immune pathogenesis and target antigen. Our research group previous discovered that FTL might be the target antigen of IRP through SEREX method and then found that serum of patients with IRP have an increase in FTL antibodies through indirect ELISA. In this study, we found that the level of FTL on the membrane of BMMNCs was significantly increased (*P* < 0.05). Compared with the case-control group, the level of FTL mRNA and FTL protein in untreated group was significantly increased by Q-PCR and WB method separately. We conclude that FTL is the target antigen of anti-BM cell membrane antibody in some patients with IRP, and it is related to the severity of disease. The mechanism of FTL as target antigen is that abnormal expression of FTL may exist in some patients with IRP, that is, FTL appears on the membrane surface that should not appear originally and acts as target antigen to cause autoimmune-mediated blood cells destruction. Our further study found that the level of FTL was increased on the surface of CD34^+^ BM cells in patients with IRP but normal in CD235a^+^ BM cells and CD15^+^ BM cells, indicating that autoantibodies attack hematopoietic stem/progenitor cell in patients with IRP.

Epitopes are specific chemical groups that determine antigen specificity. We screened and synthesized the possible FTL antigen peptide. We found that VNLYLQASYTYLSLG could activate Th2 cells, but not Th1 cells, indicating that this peptide may be the possible epitope of the target antigen in patients with IRP.

Ferritin consists of two subunits: FTL and FTH. The ratio of heavy chain and light chain varies in different tissues or special environments, which can be dynamically adjusted. Previous studies have shown that FTH has an immune-regulating effect and plays an important role in the pathogenesis of autoimmune diseases. Gray et al. proposed that FTH pretreated monocyte-derived dendritic cells and found that the relative expression of CD86 and B7-H1 on their surface was increased. When co-cultured with regulatory T cells, IL-10 production was significantly increased. These indicated that FTH activated regulatory T cells to produce IL-10 by influencing the molecular expression on the surface of dendritic cells. Monoclonal antibodies against CD86 and B7-H1 receptors, respectively, inhibit IL-10 production by regulatory T cells (28). Another study showed that FTH downregulation leads to a reduction of MHC class I molecules on the surface of macrophages. Conversely, in NCOA4-deficient mice, impaired phagocytic ferritin function leads to intracellular accumulation of FTH and MHC class I overexpression. Therefore, the reduction of FTH may affect the expression of MHC class I molecules that lead to NK cell activation (29). Mewar et al. (30) isolated the cDNA clone of FTH peptide by SEREX method and verified the existence of anti-ferritin antibody in patients with rheumatoid arthritis (RA) by the ELISA method. The ferritin antibody not only appeared in the early stage of the disease but also correlated with the severity of radiation damage. Nesher et al. (31), using recombinant antigen cDNA libraries and proteomics methods, found that 92% of 36 patients with giant cell arteritis (GCA) and/or polymyalgia rheumatica (PMR) had autoantibodies to human ferritin peptide (N-terminal of heavy chain).

**Figure 5:**
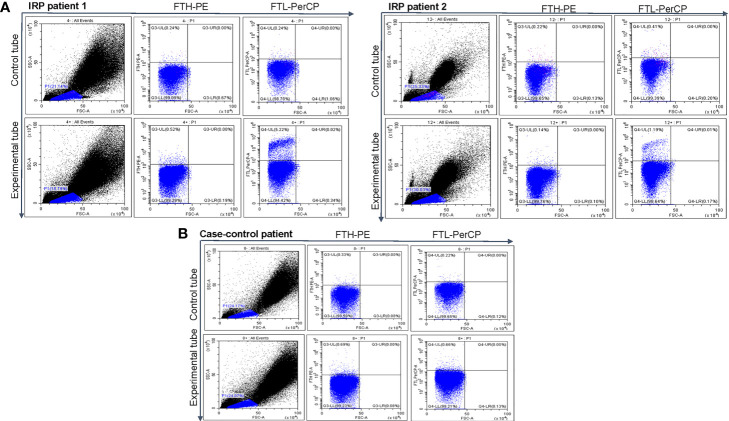
Levels of FTL and FTH. **(A)** The expression levels of FTL and FTH on BMMNCs membrane of two patients with IRP. **(B)** The expression levels of FTL and FTH on the surface of BMMNCs of one case-control group.

Our experiments have suggested that patients with IRP have elevated FTL levels. Is FTH, another component of ferritin, also elevated? Does FTH play a role in IRP abnormal immunity? Hence, we further used FCM to detect the expression levels of FTL and FTH on the surface of BMMNCs in the two patients with IRP and one case-control patient. The results showed that the expression levels of FTH on the membrane of BMMNCs from the two patients with IRP were not different from one case-control patient, but the expression level of FTL on the patient’s cell membrane was elevated (**Figure 5**). This suggested that the abnormality of ferritin on the membrane of BMMNCs in patients with IRP was mainly FTL. However, the number of cases is limited, which requires further research.

A study showed that ferritin-like structure was also found in various microorganisms, which not only used ferritin-like structure to store iron but also found that the ferritin-like protein of listeria was the target of humoral reaction in mice infected with pathogenic listeria (32). In animal models, monoclonal antibodies against ferritin-like structure can enhance the body’s resistance to listeria prior to infection, thus demonstrating the significance of upregulating the expression of cell surface related ferritin like structure *in vivo*. *In vitro* studies have shown that enhanced resistance to listeria is due to enhanced phagocytosis of listeria (33). Baerlecken et al. (34) speculated that bacterial infection may induce PMR and GCA, and anti-ferritin antibody is a response to the infection. Combined with our experimental results, we speculated that a certain microorganism contains FTL-like structure, which is the immunogen of this microorganism’s sensitized human body. However, the FTL-like structure of microorganisms has a cross-immune reaction with human FTL, which activates human autoimmunity and causes pancytopenia. Therefore, whether the peptide that we screened is only a segment of FTL or a peptide containing proteins expressed by other microorganisms still requires careful comparison and mining in future work.

In summary, we found that FTL is the target antigen for some patients with IRP. The peptide of VNLYLQASYTYLSLG is a target antigen epitope. The target antigen is abnormally overexpressed on the membrane of bone marrow BM cells, especially on the surface of CD34^+^ BM cells of patients with IRP. In addition, it is related to the severity of disease. FTL is expected to be a therapeutic target for IRP.

## Data Availability Statement

The original contributions presented in the study are included in the article/supplementary material. Further inquiries can be directed to the corresponding authors.

## Ethics Statement

The studies involving human participants were reviewed and approved by The Ethics Committee of the General Hospital of Tianjin Medical University. Written informed consent to participate in this study was provided by the participants’ legal guardian/next of kin.

## Author Contributions

SH, RF, and ZS designed the experiments. YaZ, NX, and YuZ performed the experiments. HW and LL contributed essential reagents or tools. YuZ analyzed the data. SH and YaZ wrote the manuscript. All authors contributed to the article and approved the submitted version.

## Funding

This manuscript was supported by the National Natural Science Foundation of China (81600088 and 81770118), Tianjin Health Science and Technology Project (RC 20007), and Zhao Yi-Cheng Medical Science Foundation of Tianjin (ZYYFY2019029).

## Conflict of Interest

The authors declare that the research was conducted in the absence of any commercial or financial relationships that could be construed as a potential conflict of interest.

## Publisher’s Note

All claims expressed in this article are solely those of the authors and do not necessarily represent those of their affiliated organizations, or those of the publisher, the editors and the reviewers. Any product that may be evaluated in this article, or claim that may be made by its manufacturer, is not guaranteed or endorsed by the publisher.
